# Awareness of HIV transmission, attitudes and practices amongst secondary students in an urban metropolis in Ibadan, south west Nigeria 2012

**DOI:** 10.1186/2047-2994-4-S1-P110

**Published:** 2015-06-16

**Authors:** OT Olugbade, A Aderinoye

**Affiliations:** 1 Field Epidemiology Practice, Nigeria Field Epidemiology and Laboratory Training Programme, Asokoro, Nigeria; 2 Department of Social Work, Faculty of Education, University of Ibadan, Ibadan, Oyo State, Nigeria

## Introduction

The World Health Organization estimates that of the 2.6 million new cases of Human Immunodeficiency Virus (HIV) Infections worldwide annually, nearly 370,000 (14%) occur in persons under 15 years. Nigeria has the 2nd highest number of new infections world-wide with 3.7% of the population living with HIV.

## Objectives

Our objective was to collect information on socio demographic characteristics, knowledge about prevention of transmission of HIV and Sexually Transmitted Infections (STIs), and assess the attitude towards information about subject matter, and sexual practices.

## Methods

We conducted a descriptive cross sectional survey, amongst adolescent secondary school students in Ibadan, Oyo State, Nigeria. We used a multistage sampling technique to select 270 respondents. Structured researcher-guided self-administered questionnaire was adapted to collect information. Data analyzed was expressed as descriptive statistics and chi-square (x2).

## Results

Mean age of respondents was 14.5 ± S.D 1.5 years. Of 270 students, 102 (38%) were male, 224 (83%) affirmed that sex should be delayed, however 26 (10%) of the students were sexually active, with all 26 (100%) of this proportion having unprotected intercourse. A total 124 (45.9%) believe that unprotected sex increases the risk of HIV/AIDS, sexually transmitted infections and unplanned teenage pregnancy, with knowledge being significantly associated with female gender (x2= 8.757; p<0.05) and being a senior secondary student (x2=53.758; p<0.05).

## Conclusion

Lack of information about sexual health and HIV, poor knowledge of HIV transmission and unprotected intercourse amongst sexually active adolescents, are major drivers of HIV infections, and barriers to ending the pandemic in at risk groups. Sexual health and sexuality education should be incorporated into secondary school curriculum in Oyo state, and in Nigeria, with improved access to information on safe sexual behavior through youth-oriented public awareness campaigns.

## Disclosure of interest

None declared.

